# Mapping Soil Organic Carbon by Integrating Time-Series Sentinel-2 Data, Environmental Covariates and Multiple Ensemble Models

**DOI:** 10.3390/s25072184

**Published:** 2025-03-30

**Authors:** Zhibo Cui, Songchao Chen, Bifeng Hu, Nan Wang, Chunhui Feng, Jie Peng

**Affiliations:** 1 College of Agriculture, Tarim University, Alar 843300, China; 10757232046@stumail.taru.edu.cn (Z.C.); pjzky@taru.edu.cn (J.P.); 2 Research Center of Oasis Agricultural Resources and Environment in Southern Xinjiang, Tarim University, Alar 843300, China; 3 ZJU-Hangzhou Global Scientific and Technological Innovation Center, Zhejiang University, Hangzhou 311215, China; chensongchao@zju.edu.cn; 4 College of Environment and Resources Sciences, Zhejiang University, Hangzhou 310058, China; 5 Department of Land Resource Management, Jiangxi University of Finance and Economics, Nanchang 330013, China; hubifeng@zju.edu.cn; 6 Key Laboratory of Data Science in Finance and Economics of Jiangxi Province, Jiangxi University of Finance and Economics, Nanchang 330013, China; 7 Department of Earth System Science, Ministry of Education Key Laboratory for Earth System Modeling, Institute for Global Change Studies, Tsinghua University, Beijing 100084, China; wangnanfree@zju.edu.cn; 8 College of Horticulture and Forestry, Tarim University, Alar 843300, China; 9 Key Laboratory of Tarim Oasis Agriculture, Tarim University, Ministry of Education, Alar 843300, China; 10 Key Laboratory of Genetic Improvement and Efficient Production for Specialty Crops in Arid Southern Xinjiang of Xinjiang Corps, Alar 843300, China

**Keywords:** Sentinel-2, soil organic carbon, textural information, ensemble model

## Abstract

Despite extensive use of Sentinel-2 (S-2) data for mapping soil organic carbon (SOC), how to fully mine the potential of time-series S-2 data still remains unclear. To fill this gap, this study introduced an innovative approach for mining time-series data. Using 200 top soil organic carbon samples as an example, we revealed temporal variation patterns in the correlation between SOC and time-series S-2 data and subsequently identified the optimal monitoring time window for SOC. The integration of environmental covariates with multiple ensemble models enabled precise mapping of SOC in the arid region of southern Xinjiang, China (6109 km^2^). Our results indicated the following: (a) the correlation between SOC and time-series S-2 data exhibited both interannual and monthly variations, while July to August is the optimal monitoring time window for SOC; (b) adding soil properties and S-2 texture information could greatly improve the accuracy of SOC prediction models. Soil properties and S-2 texture information contribute 8.85% and 61.78% to the best model, respectively; (c) among different ensemble models, the stacking ensemble model outperformed both the weight averaging and sample averaging ensemble models in terms of prediction performance. Therefore, our study proved that mining spectral and texture information from the optimal monitoring time window, integrated with environmental covariates and ensemble models, has a high potential for accurate SOC mapping.

## 1. Introduction

Soil organic carbon (SOC) is a key element in the global carbon cycle and plays a vital role in addressing climate change [1,2]. The global stock of SOC in arid zones is as high as 646 Pg, which exceeds the organic carbon stock of all vegetation on earth and occupies a crucial position in the global carbon sink [3]. However, as the expansion of land reclamation in arid regions expands, water scarcity has become increasingly prominent, leading to further exacerbation of aridification [4]. Against the backdrop of intensifying aridification, SOC stocks are highly susceptible to impact [5]. Therefore, high-accuracy mapping of SOC in arid zones is essential for ensuring soil health, protecting the ecological environment, managing regional ecosystem carbon sinks and achieving the ‘4 per 1000’ soil initiative [6,7,8,9].

Digital soil mapping (DSM) has been significantly enhanced by the widespread adoption of remote sensing technology, which has emerged as a critical tool for monitoring soil properties on a large scale [10]. The Sentinel-2 (S-2) satellite offers high temporal and space resolution multispectral images, which provides valuable covariate data for fine mapping of SOC at regional, national and global [11,12] levels. Currently, research on mapping SOC using S-2 data, often relying on single or multiple temporal data, primarily focuses on agricultural ecosystems in temperate regions [13]. The monitoring accuracy is generally low, with the prediction model coefficient of determination ranging from 0.02–0.62 [13,14,15]. Single temporal data represent a single data acquisition at a specific moment, providing a static view, while multiple temporal data consist of data collected at multiple discrete time points [16]. A single temporal data only captures static surface information at a specific moment, lacking the ability to represent the dynamic processes of environmental shifts and vegetation development [16]. While observations at different times enable multiple temporal data to overcome the limitations of single temporal data, their discrete nature or focus on specific periods still limit their ability to fully capture vegetation phenology dynamics or short-term variation [17]. For monitoring soil properties, research has demonstrated that time-series data generally achieve better accuracy compared to single or multiple temporal data [17]. Time-series data are a sequence of data collected over a continuous period, enabling the capture of dynamic changes [16]. Reference [18] employed 2018–2019 time-series S-2 images to map SOC distribution across croplands in Xuanzhou, Anhui Province, China. Reference [19] predicted surface SOC in cropland across soil types in the Northern Hemisphere using time-series S-2 images for 2019–2021. Time-series S-2 data provide richer temporal information than single or multiple temporal data. However, the size of these datasets can surge exponentially, occasionally growing by an order of magnitude, leading to significant computational demand. Despite the richness of these datasets, not all temporal features are relevant for monitoring SOC. Therefore, effectively utilizing the intricate time-series S-2 and extracting the most relevant temporal features remain a critical challenge in current research.

In previous DSM studies, most have primarily used spectral information as auxiliary datasets, or have mined deeply into this data to develop multidimensional spectral indices for monitoring soil properties [20,21]. However, the spatial dimensional information and geometric shapes of soil are often neglected [22]. Remote sensing images provide various spatial features, with texture features being important variables as they effectively reveal the spatial characteristics of objects [23]. By reflecting spatial variation in image brightness, texture features can reveal the unique structural information of different land cover types, thereby providing a more intuitive depiction of spatial information in images [11]. Research has demonstrated that incorporating texture features as auxiliary variables in DSM can improve the accuracy of soil property monitoring [23]. Reference [22] improved soil type mapping accuracy by combining multiple temporal Landsat 8 spectral indices with texture features. Reference [20] improved soil salinity prediction model accuracy by combining Gaofen-2 spectral indices with texture features. However, similar attempts on SOC have rarely reported on using texture features for monitoring SOC. SOC is a key factor influencing the spatial configuration of the soil’s surface structure, with its heterogeneity reflected in the variation in image pixel greyness in remote sensing images and expressed through distinct texture features [11,22]. Therefore, texture features can provide valuable information for SOC prediction. Mining S-2 texture features and developing multidimensional texture indices have a promising future in high-accuracy SOC mapping.

The choice of environmental covariates is critical to the high accuracy of SOC mapping [24]. Topography is essential for soil formation and significantly affects the spatial distribution of surface SOC [6]. It has become a commonly used environmental covariate for SOC mapping [25]. Reference [10] reported that nearly 50% of SOC mapping studies used spectral information combined with topography as covariates and the combination of spectral information and topography has become a routine covariate for SOC prediction. The frequency of application of the SCORPAN factor is usually constrained by the accessibility of environmental covariates [25]. Therefore, soil properties that influence SOC are often neglected. These properties significantly affect the processes of SOC decomposition and accumulation, so the available information on soil layers is an important basis for highly accurate SOC mapping [26]. Reference [27] included 15 soil properties to predict SOC in agricultural fields in Jiangxi Province, China. Reference [28] showed that adding soil properties could enhance the precision of SOC prediction models. Thus, using open-source soil properties data as auxiliary variables can improve the accuracy of SOC mapping. For example, as a global open-source soil property database, SoilGrids has great potential for SOC mapping and holds significant global value for broader applications [27]. However, the time-series S-2 data and environmental covariates still contain relatively rich feature variables. Here, feature selection algorithms are important for dealing with this challenge [27]. Among the many feature selection algorithms, Boruta can identify the most relevant features and remove redundant variables, thereby improving the accuracy of SOC mapping [29].

The predictive model is another crucial aspect of DSM research [24]. Current research has primarily explored single models, particularly deep learning and machine learning methods [10]. By integrating the outputs of multiple base learners, ensemble models improve generalization and robustness, demonstrating strong potential for DSM applications [27]. For instance, Reference [30] applied a stacking ensemble model to map soil moisture in Ningxia, China, while Reference [28] utilized a weight averaging ensemble model to estimate SOC in northern Iran’s forests. Similarly, Reference [26] employed the weight averaging ensemble approach to map SOC density across China. Nevertheless, the comparative performance of different ensemble models in SOC mapping remains unexplored.

Therefore, this study seeks to explore the capability of time-series S-2 data for digital SOC mapping, by developing a high-accuracy SOC mapping strategy that integrates time-series S-2 data, environmental covariates and ensemble models. The primary goals of this study are threefold: (a) to develop a novel idea for mining the important temporal features of time-series S-2 data for mapping SOC; (b) to explore the capability of S-2 texture information and soil properties for enhancing SOC mapping; (c) to assess the comparative effectiveness of different ensemble models in SOC mapping.

## 2. Materials and Methods

### 2.1. Study Area and Soil Sample Collection

**Figure 1:**
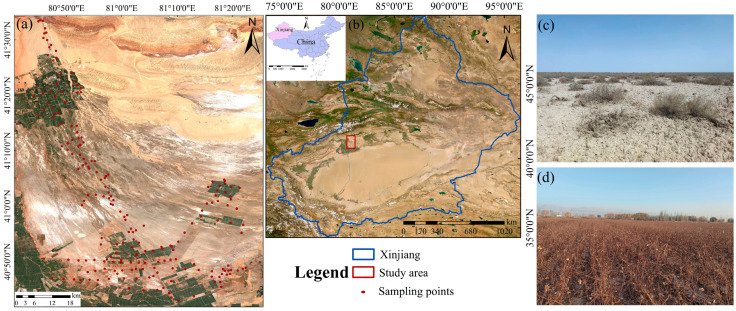
Study area overview: (**a**) spatial distribution of soil sampling locations; (**b**) Study area location; (**c**) desert region; (**d**) cotton field.

Our study site is situated in Aksu Prefecture, in the southern region of Xinjiang, China, which features a continental arid climate (Figure 1). The study area experiences a characteristic temperate continental arid climate, with the ratio of annual evaporation to precipitation close to 30 (1948 mm year^−1^:65.4 mm year^−1^) [4]. It has lower elevations to the south and higher elevations to the north (993–2121 m). A substantial volume of sediment transported by the Tianshan snowmelt has deposited at the mountain’s outlet, giving rise to a characteristic alluvial fan. Soil types mainly include meadow saline soils and brown desert soils, with high degrees of salinization and sand content. The vegetation types mainly include natural species such as *Halocnemum strobilaceum*, *Tamarix chinensis* Lour and *Haloxylon ammodendron*, as well as salt-tolerant crop cotton. In recent years, various degrees of desert areas have been converted into cropland to address the increasing food demands of a growing population. The continuous expansion of agricultural activities has profoundly impacted soil aggregate stability and SOC stocks, while also posing a threat to local agricultural resources and the ecological environment. In 2021, we collected 200 samples from the 0–20 cm topsoil layer along the main and secondary roads using a mixed sampling method and ensured that the landscape characteristics were consistent within the sampling area of each mixed sampling point individually. After laboratory pretreatment, SOC was analyzed using the externally heated potassium dichromate oxidation method [17]. The findings showed that SOC content varied between 0.74 g kg^−1^ and 13.41 g kg^−1^. The average SOC content was 4.82 g kg^−1^, with a standard deviation (SD) of 2.50 and a coefficient of variation of 51.86%. These measured SOC values were used to construct a ground-reference dataset for training and validating the SOC mapping models in this study.

### 2.2. Data Sources and Processing

#### 2.2.1. Topographic Data

The digital elevation model utilized in our study is the 30 m resolution SRTM product provided by NASA. We calculated a total of 10 topographic variables, including the following: elevation, longitudinal curvature, aspect, valley depth, slope, curvature, flow direction, topographic wetness index, convergence index and channel network base level (Table 1). In ArcGIS 10.8.1, the nearest neighboring method was employed to resample topographic variables to a 10 m resolution.

#### 2.2.2. Soil Properties Data

The soil properties data used in this study were obtained from SoilGrids 2.0, a global dataset with a 250 m resolution, freely provided by the International Soil Information Center. We downloaded a total of 11 soil properties, including the following: Vol. water content at −33 kPa, nitrogen, coarse fragments, sand, silt, Vol. water content at −10 kPa, clay, cation exchange capacity (at pH 7), pH water, bulk density and Vol. water content at −1500 kPa (Table 1). In ArcGIS 10.8.1, the nearest neighboring method was employed to resample the soil properties data to a 10 m resolution.

#### 2.2.3. Time-Series S-2 Data

The S-2 satellite is equipped with a multispectral imager featuring 13 spectral bands and spatial resolutions of 10 m, 20 m and 60 m [12]. In this study, we acquired imagery from 2017 to 2021 through the S2_HARMONIZED dataset on the Google Earth Engine platform. To correct atmospheric effects, the Semi-Automatic Classification Plugin Atmospheric Correction tool was applied, while cloud masking was conducted with the QA60 mask band [31]. To mitigate the impact of weather conditions on a single temporal S-2 image, monthly median composites free of clouds were generated, with all spectral bands resampled to a 10 m resolution [32]. Although the S2_SR_HARMONIZED dataset provides Level-2A S-2 imagery, it lacks complete global coverage from 2017 to 2018, including our study area. Therefore, this dataset was not used in the study. This study calculated 28 spectral indices, including triangle vegetation index (TVI), redness index (RI), brightness index (BI) and enhanced vegetation index (EVI), among others. After performing collinearity analysis and assessing correlation strength, 10 spectral indices were selected (Table 2).

The study employed the Grey Level Co-occurrence Matrix with a 3 × 3 window size, to extract 8 texture features from 10 S-2 spectral bands (excluding Band 10-SWIR-Cirrus, Band 9-Water vapor and Band 1-Coastal aerosol). The advantage of a 3 × 3 window size is that it can capture the heterogeneity of pixel values within a small area [11]. Based on existing empirical formulas for spectral indices, 3 two-dimensional and 3 three-dimensional texture indices were developed (Table 2).

This study proposed a new idea for mining time-series data. It emphasizes the temporal variation in the correlation between soil properties and time-series data [33]. The variation in the correlation between SOC and time-series data arises from the differing relationships between SOC and the data at each time point. Specifically, correlation analysis was conducted to explore temporal variation patterns between SOC and time-series S-2 data. At the monthly scale, the relationship between SOC and S-2 data exhibits significant shifts, indicating that annually, there exists a specific month where the correlation between SOC and S-2 data reaches its peak. This month was considered the optimal monitoring month for SOC. However, because of variations in the rainy season’s timing or annual differences in plant phenology, the optimal monitoring month can vary between years. This range of variation was termed the optimal monitoring time window for SOC. By determining the optimal monitoring time window, we can eliminate irrelevant temporal features, thereby improving the efficiency of SOC monitoring.

### 2.3. Scenario Construction

This study integrated spectral indices, terrain variables, texture indices and soil properties to construct four scenarios (Table 3). Building upon the conventional modeling approach that combines spectral indices with terrain variables, we assessed the improvements in SOC prediction models by adding soil properties, texture indices and a combination of soil properties and texture indices. Finally, the contribution of these four types of variables to model performance was analyzed.

### 2.4. Feature Selection Algorithm

Boruta is a feature selection algorithm that leverages Random Forest to identify significant features by comparing them with randomized shadow features [32]. By using the ggplot2, Boruta and randomForest package in R Studio for feature variable selection on Scenario A, Scenario B, Scenario C and Scenario D, a total of 40, 42, 43 and 45 variables were selected, respectively. Compared with the original dataset, the quantity of the data was reduced by 63.64%, 65.29%, 78.50% and 78.72%, respectively.

### 2.5. Modeling Approaches and Performance Evaluation

This study utilized five base learners, classified into different categories: Partial least squares regression (PLSR) represented the linear regression model, while machine learning models included gradient eXtreme gradient boosting (XGBoost), boosting regression tree (GBRT) and random forest (RF). The deep learning approach was Multilayer Perceptron (MLP). To determine the best parameter combinations, GridSearchCV was applied for hyperparameter tuning (Table 4). Utilizing 10-fold cross-validation, the performance of three ensemble techniques—stacking, weighted averaging and simple averaging—was assessed. The evaluation metrics included the coefficient of determination (R^2^), mean absolute error (MAE) and root mean square error (RMSE). Quantifying model uncertainty is vital, as it enhances understanding of forecast reliability by analyzing the SD of cross-validation predictions, complementing predictive performance assessment [34].

## 3. Results

### 3.1. Correlation Analysis of Covariates with SOC

#### 3.1.1. Correlation Between SOC and S-2 Texture Features

**Figure 2:**
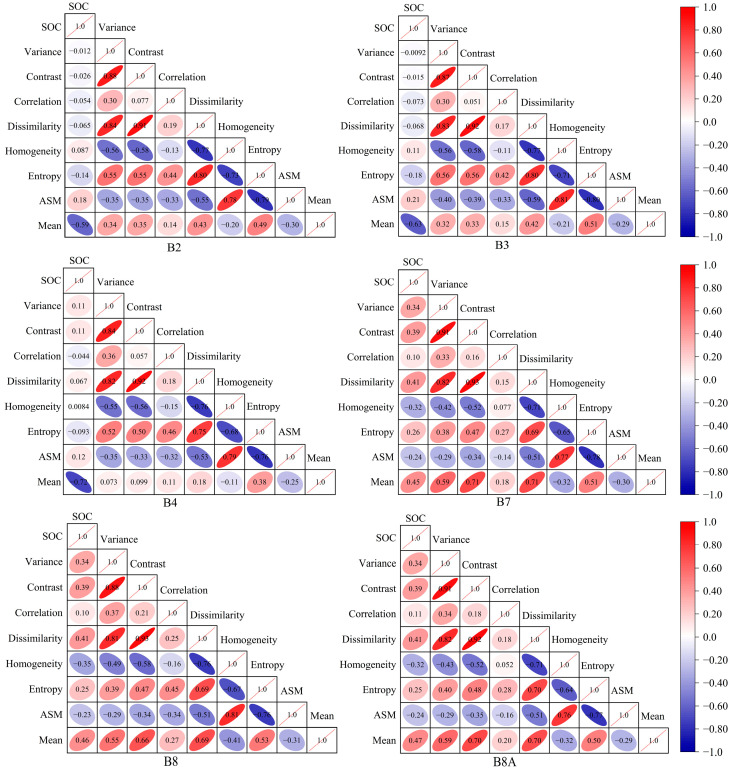
The correlation heatmaps between SOC and texture features of the 10 S-2 bands.

**Figure A1:**
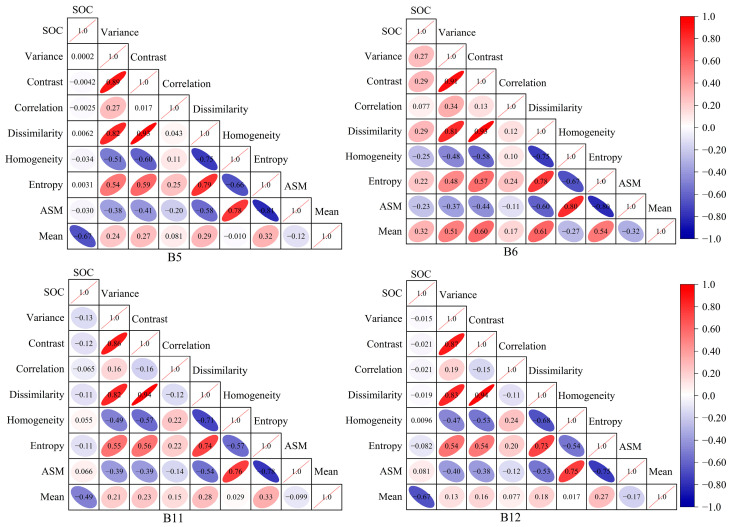
The correlation heatmaps between SOC and texture features of the 10 S-2 bands.

Figure 2 and Figure A1 show the correlation between SOC and eight texture features extracted from 10 bands of a single temporal S-2 image. Among different texture features, the correlation between SOC and Mean is higher than other texture features in all the bands and the highest value of correlation coefficient is 0.72. For texture features in different bands, the correlation between SOC and texture features derived from Band 7 (B7), B8 and B8A is significantly higher than that of other bands. In summary, the texture feature Mean had the best correlation with SOC and the texture features extracted on the B7, B8 and B8A bands had an overall better correlation with SOC. Therefore, we selected texture feature Mean on all the bands and all texture features on bands B7, B8 and B8A to develop the multidimensional texture indices.

**Figure 3:**
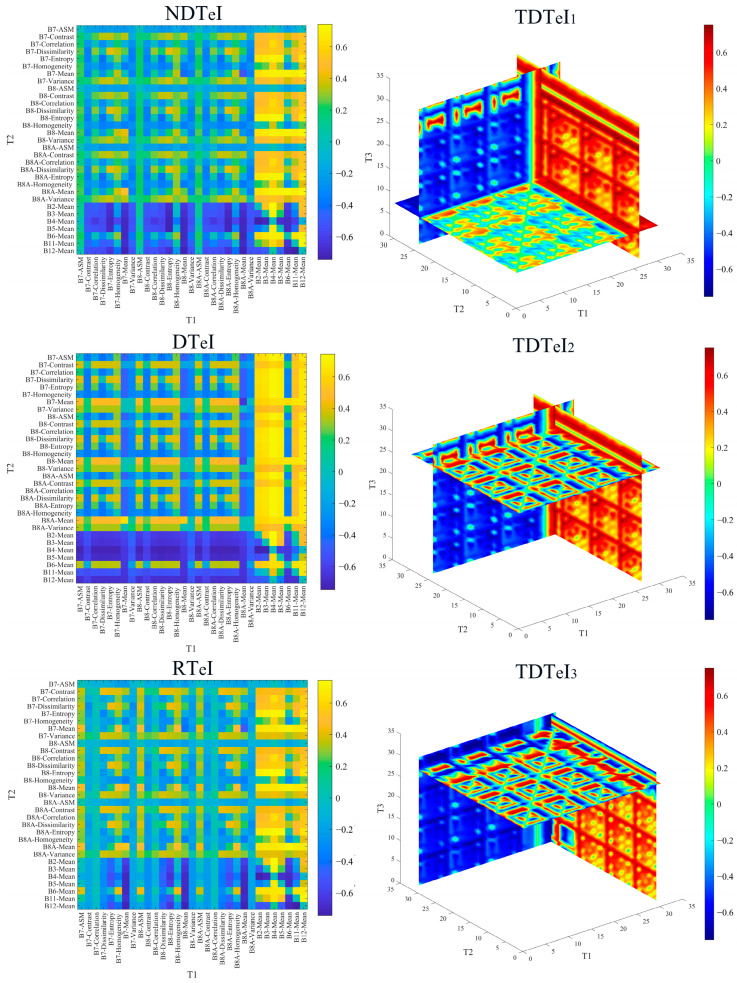
The correlations between SOC and the newly developed multi-dimensional texture indices.

Figure 3 shows the correlation between SOC and the newly developed multidimensional texture indices. The multidimensional texture indices could greatly enhance the correlation between SOC and texture features. The correlation coefficients of the best combination of two-dimensional texture indices DT_e_I, NDT_e_I and RT_e_I with SOC were 0.77, 0.74 and 0.73, respectively, which were higher than the maximum value of correlation coefficients of the one-dimensional texture features with SOC of 0.72 and the T_1_ and T_2_ in the optimal combinations were both B2-Mean and B4-Mean. The correlation coefficients of the best three-dimensional texture indices, TDT_e_I_1_ and TDT_e_I_2_, were higher than the maximum value of correlation coefficient between one-dimensional texture features and SOC, which was 0.72. TDT_e_I_3_ was lower than the maximum value of 0.72, and the correlation did not improve, so we did not consider TDT_e_I_3_ in the subsequent study. The T_1_, T_2_ and T_3_ in the optimal combination of three-dimensional texture indices TDT_e_I_1_ and TDT_e_I_2_ were B2-Mean, B4-Mean, B6-Mean and B2-Mean, B3-Mean, B4-Mean, respectively. Therefore, the two-dimensional texture indices of DT_e_I, NDT_e_I and RT_e_I and the three-dimensional texture indices of TDT_e_I_1_ and TDT_e_I_2_ could improve the correlation between SOC and texture features. We performed further mining of time-series data for these five multidimensional texture indices and the texture features involved in the development of these multidimensional texture indices.

#### 3.1.2. Correlation Between SOC and S-2 Texture Indices

**Figure 4:**
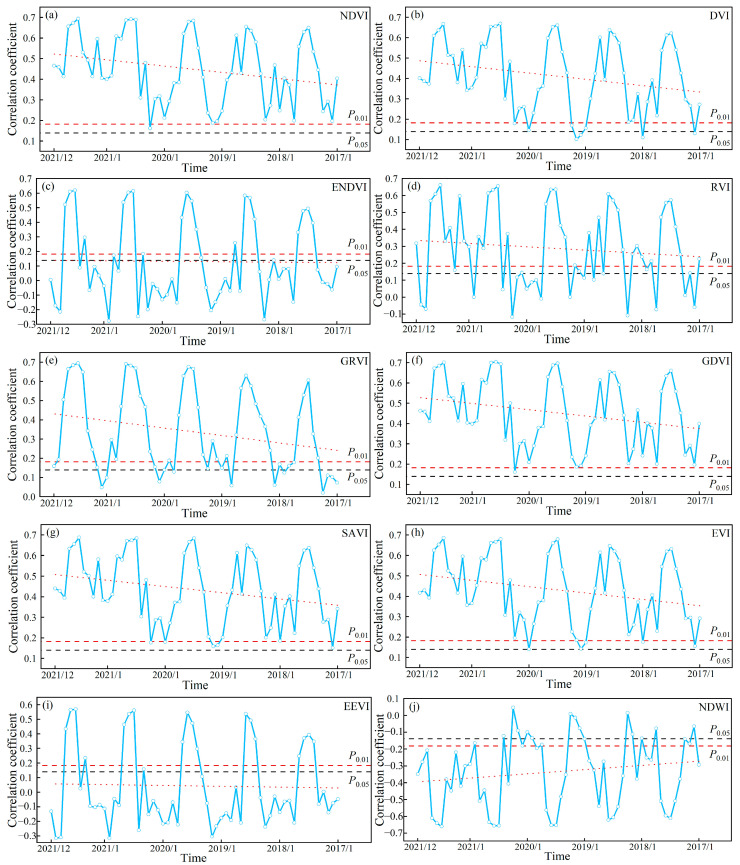
Patterns of change between SOC and time-series S-2 spectral indices. (**a**–**j**) represent the correlation changes between SOC and different time-series S-2 spectral indices. Note: The black solid line denotes the correlation coefficient at a significant level (*P*_0.05_ = 0.139), whereas the red solid line denotes the correlation coefficient at a highly significant level (*P*_0.01_ = 0.182). The red dashed line shows the overall trend between SOC and time-series S-2 spectral indices.

**Figure 5:**
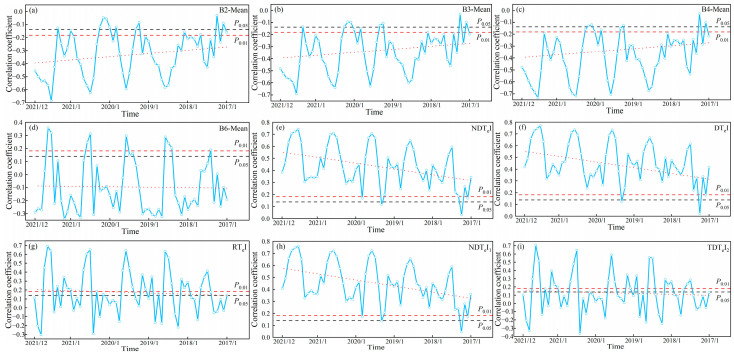
Patterns of change between SOC and time-series S-2 texture indices. (**a**–**i**) represent the correlation changes between SOC and different time-series S-2 texture indices. Note: The red dashed line shows the overall trend between SOC and time-series S-2 texture indices.

Figure 4 and 5 show the temporal variation pattern of correlation between SOC and time-series S-2 data. The correlation between SOC and time-series S-2 data exhibited an annual cyclic variation. As shown in Figure 4, the correlation between SOC and time-series S-2 spectral indices showed an increase followed by a decrease from January to April, a similar pattern from May to September, and a continuous increase from October to December. From Figure 5, the correlation between SOC and time-series S-2 texture index showed an increase followed by a decrease from January to April, and a similar pattern from May to December. Thus, throughout the annual cycle, there was always a month when the correlation between SOC and time-series S-2 data peaked, defining the optimal monitoring month for SOC. However, the optimal monitoring month varied between years, with a concentration in July and August. We determined July and August as the optimal monitoring time window for SOC. Identifying the optimal monitoring time window led to an 83.33% reduction in data volume relative to the initial time-series dataset.

The interannual variation pattern revealed a gradual decline in the correlation between SOC and time-series S-2 data, with the rate of decrease varying across different indices. However, because of the time limitations of the time-series S-2 data, the correlation between SOC and the data within the optimal monitoring time window remained strongly significant. Nevertheless, the maximum valid monitoring year for SOC has not yet been identified.

### 3.2. Assessment and Comparison of Multiple Scenarios by Different Ensemble Models

Table 5 displays the results of multiple ensemble models developed based on four distinct scenarios. The results indicated that the choice of scenario and ensemble model significantly impacted modeling accuracy. First, in terms of the comparison among different ensemble models, the stacking ensemble model outperformed weight averaging and simple averaging ensemble models. Second, adding soil properties and texture indices enhanced the predictive capability of the models. Compared to Scenario A, adding soil properties in Scenario B increased R^2^ by 0.02, and decreased MAE and RMSE by 0.06 g kg^−1^ and 0.07–0.08 g kg^−1^, respectively. Similarly, compared to scenario A, adding texture information in Scenario C increased R^2^ by 0.04–0.05, and decreased MAE and RMSE by 0.12 g kg^−1^ and 0.13–0.14 g kg^−1^, respectively. Finally, Scenario D, which added both soil properties and texture information, attained the greatest accuracy among the three ensemble models. Compared to Scenario A, Scenario D showed an increase in R^2^ by 0.06–0.07, and a decrease in MAE and RMSE by 0.18 g kg^−1^ and 0.19 g kg^−1^, respectively.

### 3.3. The Significance of Feature Variables

**Figure 6:**
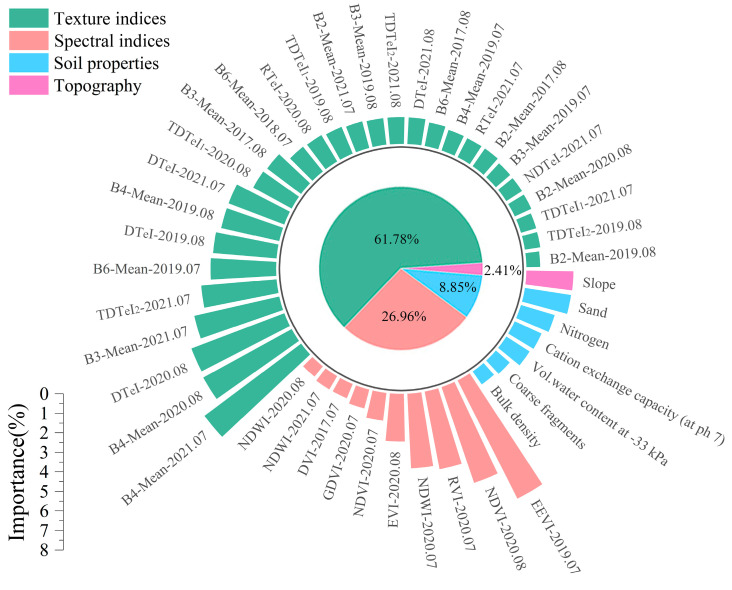
The significance of variables in the optimal predictive model.

Figure 6 displays the contribution of each feature variable to the optimal predictive model. Normalizing feature variable importance to a total of 100% improved comparability, providing a more accurate depiction of their relative roles in the SOC prediction model. B4-Mean-2021.07 was the most important individual variable, with a relative importance of 6.17%. NDWI-2020.08 made the least contribution, representing just 0.68% of the total impact. Among similar feature variables, texture indices exhibited the highest relative importance, followed by spectral indices and soil properties, whereas topographic factors contributed the least. Among the texture indices, the multidimensional texture indices had a relative importance of 28.96%, indicating a significant contribution to the optimal predictive model. Among the soil properties, sand was the most important. Topography showed the lowest contribution among all the variables, reflecting the flat terrain and minimal influence of topographic factors on SOC patterns in the region. Most feature variables were concentrated in years closer to the sampling period (2019–2021), while fewer were selected from 2017–2018, as indicated by their temporal distribution. This pattern aligns with the observed correlation trend between SOC and time-series S-2 data.

### 3.4. Spatial Distribution of SOC and Its Uncertainty

**Figure 7:**
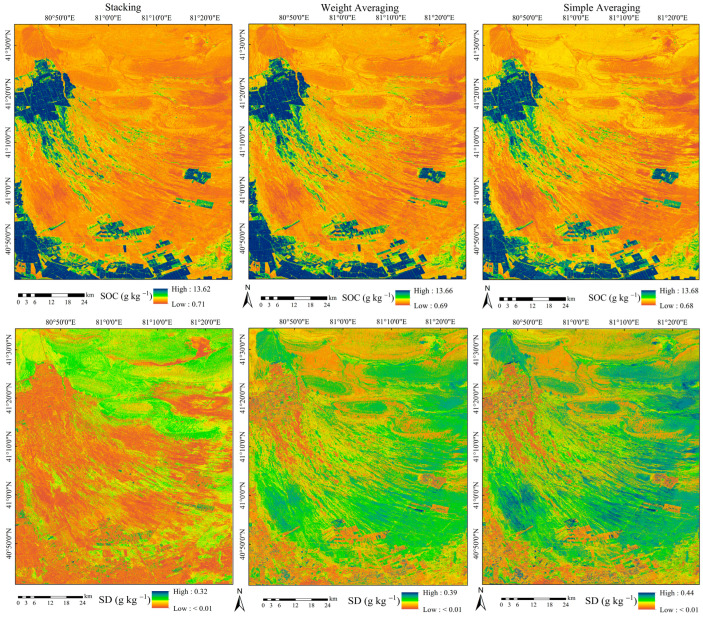
Predicted SOC (g kg^−1^) and SD (g kg^−1^) distributions derived from three ensemble models.

Figure 7 illustrates the predicted SOC distribution along with the associated uncertainty, based on the three ensemble models derived from Scenario D. The SOC distribution trends predicted by the three ensemble models were generally consistent. SOC displayed significant spatial heterogeneity, with long-term cultivated farmland exhibiting higher SOC levels, while newly cultivated fields, desert regions, and mountainous areas had lower SOC content. Sustained fertilization and the practice of returning straw in long-term cultivated fields contributed to an enhanced supply of SOC, leading to higher levels. In contrast, desert and mountainous regions had lower SOC levels, primarily because of limited vegetation cover, which resulted in insufficient SOC inputs. Newly cultivated farmland, originally part of the desert, inherently had low SOC content, and due to the short cultivation period, the enhancement of soil fertility has been constrained and remains relatively limited. Despite the three ensemble models producing similar overall SOC distribution patterns, significant variations were evident in specific spatial details. In the northern mountainous and desert regions, the simple averaging and weight averaging ensemble models tended to overestimate low SOC areas compared to the stacking ensemble model. The predicted mean SOC values for the three ensemble models were 4.76 g kg^−1^, 4.68 g kg^−1^ and 4.62 g kg^−1^, respectively. The stacking ensemble model provided SOC predictions with a mean and range that were most similar to the ground survey data, outperforming both the simple averaging and weight averaging ensemble models. As a result, the stacking ensemble approach outperformed other methods in SOC prediction and effectively captured the overall spatial distribution pattern.

Digital SOC mapping inherently involves a certain degree of uncertainty. The three ensemble models exhibited a largely consistent pattern in SOC uncertainty distribution. SOC uncertainty was generally higher in mountainous and desert regions with low SOC content, whereas areas like farmland, where SOC levels were higher, exhibited lower uncertainty. The stacking ensemble model exhibited a lower average SOC uncertainty (0.20 g kg^−1^) compared to the weight averaging ensemble model (0.26 g kg^−1^) and the simple averaging ensemble model (0.29 g kg^−1^). Furthermore, the stacking ensemble model effectively reduced SOC uncertainty in areas where high uncertainty persisted in the simple averaging and weight averaging models. The heightened uncertainty in desert and mountainous regions stemmed primarily from the scarcity of sampling points, a consequence of challenging terrain and limited accessibility.

## 4. Discussion

### 4.1. Enhancing SOC Mapping with Time-Series S-2 Data

The effectiveness of remote sensing technology in predicting SOC relies on both the accessibility and quality of the imagery [13]. Compared to time-series images, both single temporal and multiple temporal images are more susceptible to the influence of weather conditions [32]. Moreover, the ability to predict SOC by analyzing soil-vegetation relationships relies on the capture of vegetation characteristics that reflect changes in SOC through remote sensing imagery [35]. However, SOC content is influenced not only by the vegetation data of the current year but also by the vegetation conditions of previous years [36]. This is because SOC represents the accumulation and decomposition of plant litter over several years in the soil [27]. As a result, long-term vegetation data have a more significant impact on SOC predictions than short-term vegetation data. Time-series images can track the continuous dynamic changes in vegetation, thus providing a more effective way to monitor SOC fluctuations over time [6]. However, redundant information in time-series data can lead to higher computational complexity, ultimately reducing model efficiency and performance. To overcome this challenge, the study introduces a time-series data mining method that determines the optimal monitoring time window and the maximum valid monitoring year for SOC by analyzing the temporal changes in the relationship between SOC and time-series S-2 data. Through feature variable selection, the most relevant temporal features for SOC monitoring were identified. The primary objective of our study is to identify the optimal monitoring time window by examining the periodic correlation trends between SOC and time-series S-2 data, subsequently extracting critical temporal features to facilitate the efficient use of complex time-series data.

Most research using satellite remote sensing for SOC monitoring and mapping has predominantly relied on either single or multiple temporal data, yet the precision of these assessments often fluctuates based on the specific timing of the observations made [19]. Identifying the optimal monitoring time window for SOC is crucial when utilizing single or multiple temporal data, as it offers a theoretical basis for choosing the most suitable period for acquiring remote sensing data and acts as a useful guideline for analogous areas. Some studies have also investigated the optimal monitoring period for soil property. Reference [37] analyzed the accuracy of soil organic matter (SOM) predictions during the bare soil period, revealing that the most reliable results were obtained in mid-May in China’s Songnen Plain. Reference [32] assessed SOM prediction accuracy from April to October, identifying the April–June period as yielding the highest accuracy. However, these studies primarily considered the annual scale, overlooking the year-to-year fluctuations in the optimal monitoring period caused by phenological variations. Unlike previous studies, our research investigated the optimal SOC monitoring window by tracking temporal variations over multiple years, allowing for a more precise assessment of both seasonal and interannual SOC dynamics. For southern Xinjiang, China, the optimal monitoring time window for SOC was identified as July–August. This period corresponds to the wet season and the peak vegetation growth phase in the study area. The wet season significantly increases precipitation, leading to a substantial rise in soil moisture. The region’s dry climate and high evaporation rates lead to salt buildup on the soil surface, which subsequently affects the soil’s spectral reflectance [4]. However, during periods of increased rainfall, the elevated precipitation facilitates the downward movement of salts into deeper soil layers, thereby diminishing their influence on the spectral reflectance associated with SOC [4]. During this time, different plant species reach their peak growth stages, and it is the period when maximum biomass is produced across various plants in this region. Given the essential role of SOC in plant growth, S-2 indices effectively capture canopy characteristics during peak vegetation periods, enhancing SOC monitoring [17].

**Figure 8:**
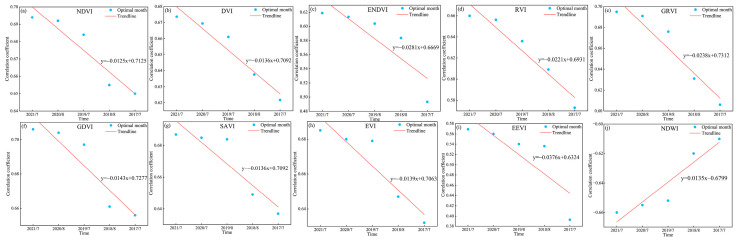
The correlation between SOC and the optimal monitoring month of spectral indices. (**a**–**j**) show the variation trends of correlations between SOC and different spectral indices across optimal monitoring months, respectively.

**Figure 9:**
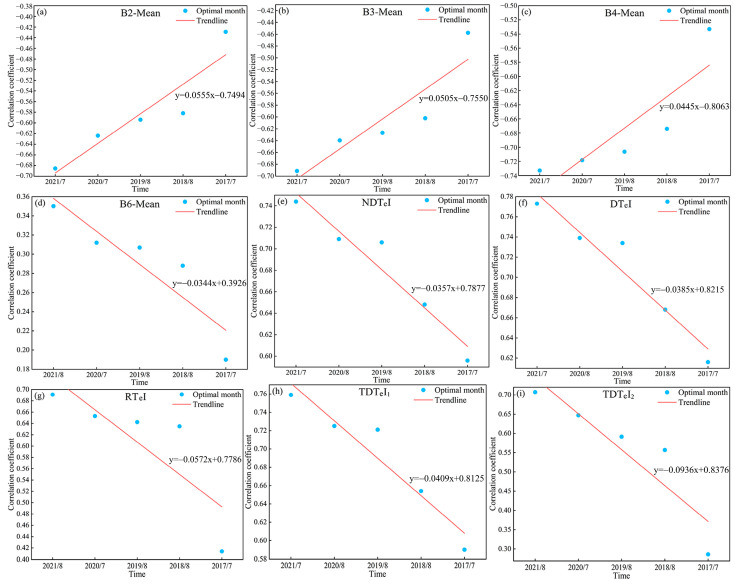
The correlation between SOC and the optimal monitoring month of texture indices. (**a**–**i**) show the variation trends of correlations between SOC and different texture indices across optimal monitoring months, respectively.

Although we did not directly mind the maximum valid monitoring year for SOC using time-series S-2 data, the decreasing trend in the correlation between SOC and data from the optimal monitoring time window allowed us to infer the maximum valid monitoring year for SOC. Figure 8 and Figure 9 show the decreasing trend line of the optimal monitoring month correlation with SOC. Based on the decreasing trends of different indices, it can be inferred that the maximum valid monitoring year for SOC using S-2 spectral indices ranges from 13 to 49 years, while for texture indices, it ranges from 7 to 18 years.

### 4.2. The Importance of Mining S-2 Texture Information for Mapping SOC

In digital SOC mapping, it is crucial to identify remote sensing indicators related to SOC [24]. This is vital in arid regions, where highly salinized soil surfaces can lead to salt patches or salt crusts, weakening the soil’s spectral characteristics and resulting in the ‘different objects, same spectra’ and ‘same object, different spectra’ phenomena [23]. Therefore, it poses a major challenge for SOC mapping studies that rely only on spectral features.

Remote sensing images can provide abundant and distinct spatial information [10]. Texture features can reflect the structural feature and spatial variation in grey values in images, providing supplementary information for image attributes and thus compensating for the lack of soil spectral information [23]. By fully using the structural information of image grey distributions, differences between various land cover types can be enhanced, reducing confusion caused by mixed pixels and better characterizing the spatial distribution of the soil surface [22,23]. The results of our study indicated that adding texture information improved the accuracy of SOC prediction models. R^2^ increased by 0.04–0.05, while MAE and RMSE decreased by 0.12 g kg^−1^ and 0.13–0.14 g kg^−1^, respectively. In the optimal model, texture information contributed 61.78% of the overall relative importance. These results are consistent with previous studies [22,23]. Compared with previous studies, our study not only mined the potential of temporal texture features in SOC monitoring but also systematically compared the correlation between SOC and texture features in different bands of S-2. Our results indicated that the three red-edge bands, B7, B8 and B8A, were the sensitive bands for texture features to monitor SOC, and Mean was the sensitive feature. Consistent with the finding of [38], they found that the texture features extracted from the red-edge bands were more strongly correlated in monitoring aboveground biomass of potato. Since SOC is directly related to aboveground biomass and represents a cumulative result of vegetation, apoplectic material accumulated and humified in the soil through the years [27]. The sensitive bands and features identified offer a theoretical foundation and reference for monitoring SOC using S-2 texture features. Currently, research on monitoring land cover using texture information typically relies on single texture features and has not fully used the texture information in images. The multidimensional texture indices allow for the integrated application of various texture features, enabling the effective exploration of their combined potential and enhancing the precision of SOC monitoring. The multidimensional texture indices we developed enhanced the correlation between SOC and texture features and accounted for 28.96% of the relative importance. By identifying sensitive bands and developing multidimensional texture indices, a more comprehensive reflection of SOC spatial distribution can be achieved.

### 4.3. Evaluating the Predictive Capability of Different Ensemble Models for SOC

By integrating the strengths of multiple base learners, ensemble models significantly enhance the reliability and precision of SOC prediction [26]. Our findings indicate that the ensemble model achieves a notable improvement in accuracy over individual base learners, aligning with findings from earlier studies [28,39]. Furthermore, most prior studies have primarily focused on ensemble machine learning models [30,39]. Unlike these studies, our research not only incorporated ensembles of deep learning, machine learning, and linear regression models, but also conducted a comparison among various ensemble approaches. The findings indicated that the stacking ensemble model outperformed the others, with the weighted averaging approach ranking second, whereas the simple averaging method showed comparatively lower performance.

The variations in accuracy among different ensemble models can likely be attributed to their underlying ensemble strategies. By averaging the outputs of multiple base learners, the simple averaging ensemble model enhances prediction accuracy, addressing challenges such as the underestimation of high values and overestimation of low values, thereby lowering overall prediction variance [40]. This model serves as a basic benchmark for evaluating the performance of more complex ensemble methods. Using ‘R^2^ normalization’, the weighted averaging ensemble model assigns specific weights to individual base learners, combining their outputs by multiplying predictions with their assigned weights and summing the results to generate the final prediction [26]. On the other hand, the stacking ensemble model employs cross-validation to merge the predictions of various base learners, utilizing a meta-learner to integrate these outcomes [30]. This layered approach captures complex relationships and overcomes the limitations of individual models, boosting accuracy and generalization. In contrast to simple averaging and weight averaging, the stacking model benefits from its layered structure to enhance performance. The relationship between SOC and covariates can be influenced by factors such as study area, type of remote sensing data, and environmental conditions used. Additionally, differences in dataset size and sample distribution across regions make it unlikely for any single model to perform optimally in all scenarios [24]. Future studies could refine ensemble models by incorporating additional base learners and layers to improve their generalization capability and forecasting accuracy.

## 5. Conclusions

This study used time-series S-2 data, environmental covariates, and multiple ensemble models to create a digital SOC map for the arid regions of southern Xinjiang, China. Our results showed that: (a) the optimal monitoring time window for SOC using time-series S-2 data is July–August, and the maximum effective year is inferred to be 7–49 years; (b) the sensitive bands for monitoring SOC using S-2 texture features are B7, B8 and B8A, and the sensitive feature is Mean. The newly developed multidimensional texture indices not only improve the correlation between SOC and texture features but also accounted for 28.96% relative importance in the optimal model; (c) among the soil properties, sand is most important for the SOC prediction model. We explored the significant potential of S-2 texture features in monitoring SOC and introduced a novel approach for mining time-series data. These advancements enhance our ability to monitor SOC, which is crucial for mitigating climate change, improving soil management practices, and promoting sustainable land use strategies.

## Figures and Tables

**Table 1:** Topographic variables and soil properties utilized in this study.

Variable Categories	Variables	Sources
Topographic variables	Elevation	(https://earthdata.nasa.gov/) (accessed on 10 May 2024)
	Longitudinal curvature	
	Aspect	
	Valley depth	
	Slope	
	Curvature	
	Flow direction	
	Topographic wetness index	
	Convergence index	
	Channel network base level	
	Vol. water content at −33 kPa	
Soil properties	Nitrogen	(https://soilgrids.org) (accessed on 24 May 2024)
	Coarse fragments	
	Sand	
	Silt	
	Vol. water content at −10 kPa	
	Clay	
	Cation exchange capacity (at pH 7)	
	pH water	
	Bulk density	
	Vol. water content at −1500 kPa	

**Table 2:** Remote sensing variables utilized in this study for SOC prediction, including their acronyms, calculation formulas and corresponding references.

Variables	Predictors	Acronyms	Formulas	Reference
Spectral indices	Normalized Difference Vegetation Index	NDVI	$\left(NIR-R\right)/\left(NIR+R\right)$	[16]
	Difference Vegetation Index	DVI	NIR−R	[16]
	Enhanced Normalized Difference Vegetation Index	ENDVI	$\left(NIR+{SWIR}_{2}-R\right)/\left(NIR+{SWIR}_{2}+R\right)$	[16]
	Ratio Vegetation Index	RVI	NIR/R	[16]
	Green-Red Vegetation Index	GRVI	$\left(G-R\right)/\left(G+R\right)$	[16]
	Generalized Difference Vegetation Index	GDVI	$\left({NIR}^{2}-R^{2}\right)/({NIR}^{2}+R^{2})$	[16]
	Soil-Adjusted Vegetation Index	SAVI	$\left[1.5\times \left(NIR-R\right)\right]/\left(NIR+R+0.5\right)$	[16]
	Enhanced Vegetation Index	EVI	$\left[2.5\times \left(NIR-R\right)\right]/(NIR+6\times R-$ 7.5×B+0.5)	[16]
	Enhanced Environment Vegetation Index	EEVI	$\left[1.5\times \left(NIR-R\right)\right]/\left(NIR+R+0.5\right)$	[16]
	Normalized Difference Water Index	NDWI	$\left(G-NIR\right)/\left(G+NIR\right)$	[16]
Texture indices	Angular Second Moment	ASM	$\sum_{i=0}^{N-1}\sum_{j=0}^{N-1}P{\left(i,j\right)}^{2}$	[11]
	Contrast	CON	$\sum_{i,j=0}^{N-1}{iP}_{i,j}{\left(i-j\right)}^{2}$	[11]
	Correlation	COR	$\sum_{i,j=0}^{N-1}{iP}_{i,j}\left\{\left[\left(i-mean\right)\left(j-mean\right)\right]/\sqrt{{var}_{i}*{var}_{j}}\right\}$	[11]
	Dissimilarity	DIS	$\sum_{i,j=0}^{N-1}{iP}_{i,j}\left|i-j\right|$	[11]
	Homogeneity	HOM	$\sum_{i,j=0}^{N-1}i\left\{P_{i,j}/\left[1+{\left(i-j\right)}^{2}\right]\right\}$	[11]
	Entropy	ENT	$\sum_{i,j=0}^{N-1}{iP}_{i,j}\left(-\ln P_{i,j}\right)$	[11]
	Variance	VAR	$\sum_{i,j=0}^{N-1}p_{i,j}{\left(i-\mu_{i}\right)}^{2}$	[11]
	Mean	MEA	$\sum_{i,j=0}^{N-1}p\left(i,j\right)$	[11]
	Difference Texture Index	DTeI	$T_{1}-T_{2}$	this study
	Normalized Difference Texture Index	NDT_e_I	$\left(T_{1}-T_{2}\right)/\left(T_{1}+T_{2}\right)$	this study
	Ratio Texture Index	RT_e_I	$T_{1}/T_{2}$	this study
	Three-Dimensional Texture Index 1	TDT_e_I_1_	$\left(T_{1}-T_{2}\right)/\left(T_{1}+T_{3}\right)$	this study
	Three-Dimensional Texture Index 2	TDT_e_I_2_	$T_{1}/\left(T_{2}+T_{3}\right)$	this study
	Three-Dimensional Texture Index 3	TDT_e_I_3_	$\left(T_{1}-T_{2}\right)/\left[\left(T_{1}-T_{2}\right)-\left(T_{2}+T_{3}\right)\right]$	this study

Note: the parameters P (i, j) represent the joint probability density function of grey levels i and j in the image. T_1_, T_2_ and T_3_ in the constructed multidimensional texture index represent the texture features of different bands and T_1_ ≠ T_2_ ≠ T_3_.

**Table 3:** Different combinations of S-2 spectral indices, texture indices, topographic properties, and soil properties.

Scenarios	Variables
Scenario A	Spectral indices + Topographic
Scenario B	Spectral indices + Topographic + Soil properties
Scenario C	Spectral indices + Topographic + Texture indices
Scenario D	Spectral indices + Topographic + Soil properties + Texture indices

**Table 4:** Base learners hyperparameter range.

Base Learners	Parameters Type (Range [Start, Stop, Step])
MLP	hidden_layer_sizes [(50), (100), (50, 50), (100, 50), (50, 100)], learning_rate [0.01, 0.1, 0.01], solver: [ Adam], activation: [relu]
GBRT	min_samples_leaf [1, 10, 1], n_estimators [10, 500, 10], max_depth [1, 10, 1], learning_rate [0.01, 1, 0.01], min_samples_split [1, 10, 1]
RF	min_samples_split [1, 10, 1], max_depth [1, 10, 1], n_estimators [10, 500, 10], min_samples_leaf [1, 10, 1]
XGBoost	learning_rate [0.01, 0.1, 0.01], max_depth [1, 10, 1], n_estimators [10, 500, 10]
PLSR	n_components [1, 50, 1]

**Table 5:** Comparison of accuracy for four SOC predictive scenarios with three ensemble models.

Scenario	Stacking			Weight Averaging			Simple Averaging		
Type of Scenario	R^2^	MAE	RMSE	R^2^	MAE	RMSE	R^2^	MAE	RMSE
Scenario A	0.82	1.04	1.28	0.81	1.07	1.31	0.80	1.10	1.34
Scenario B	0.84	0.98	1.20	0.83	1.01	1.24	0.82	1.04	1.27
Scenario C	0.87	0.92	1.14	0.85	0.95	1.18	0.84	0.98	1.21
Scenario D	0.89	0.86	1.09	0.87	0.89	1.12	0.86	0.92	1.15

## Data Availability

The data that support the findings of this study are available on request from the corresponding author.
